# Unraveling the Potential of *Yarrowia lipolytica* to Utilize Waste Motor Oil as a Carbon Source

**DOI:** 10.3390/jof10110777

**Published:** 2024-11-08

**Authors:** Sílvia M. Miranda, Isabel Belo, Marlene Lopes

**Affiliations:** 1 Centre of Biological Engineering, University of Minho, Campus de Gualtar, 4710-057 Braga, Portugalibelo@deb.uminho.pt (I.B.); 2 LABBELS–Associate Laboratory, 4710-057 Braga/Guimarães, Portugal

**Keywords:** waste motor oil, *Yarrowia lipolytica*, pulse fed-batch culture, protease, microbial lipids, biodiesel

## Abstract

This study evaluated the potential of *Y. lipolytica* (CBS 2075 and DSM 8218) to grow in waste motor oil (WMO) and produce valuable compounds, laying the foundation for a sustainable approach to WMO management. Firstly, yeast strains were screened for their growth on WMO (2–10 g·L^−1^) in microplate cultures. Despite limited growth, the CBS 2075 strain exhibited comparable growth to control conditions (without WMO), while DSM 8218 growth increased 2- and 3-fold at 5 g·L^−1^ and 10 g·L^−1^ WMO, respectively. The batch cultures in the bioreactor confirmed the best performance of DSM 8218. A two-stage fed-batch strategy–growth phase in aliphatic hydrocarbons, followed by the addition of WMO (one pulse of 5 g·L^−1^ or five pulses of 1 g·L^−1^ WMO), significantly increased biomass production and WMO assimilation by both strains. In experiments with five pulses, CBS 2075 and DSM 8218 strains reached high proteolytic activities (593–628 U·L^−1^) and accumulated high quantities of intracellular lipids (1.3–1.7 g·L^−1^). Yeast lipids, mainly composed of oleic and linoleic acids with an unsaturated/saturated fraction > 59%, meet the EU biodiesel standard EN 14214, making them suitable for biodiesel production.

## 1. Introduction

Waste motor oil (WMO) is a hazardous, inflammable, and water-insoluble mixture with an oil base (70–90%) manufactured from natural gas or crude oil. This complex mixture is mostly composed of aliphatic hydrocarbons (alkanes from C16–C32 and cycloalkanes), monoaromatic and polycyclic aromatic hydrocarbons (PAH), and residual amounts of olefinic compounds [1,2]. Additionally, WMO contains persistent and hazardous heavy metals like lead, nickel, iron, barium, and zinc [3]. Due to the high number of pollutants in WMO, its inappropriate disposal has serious consequences on the ecosystems and living organisms [4]. In 2023, 63,443 tons of motor oil were produced in Portugal, but only 48% was collected and recycled or regenerated [5].

Some authors have investigated the biodegradation of WMO using bacterial strains belonging to the genera *Bacillus* [6], *Streptomyces* [2], and *Pseudomonas* [7]. By contrast, the research on the assimilation of WMO by yeasts is still in its infancy. *Candida viswanathii* KA-2011 degraded 76% of the total WMO used in the culture medium [8]. Yeasts from the genera *Meyerozyma*, *Rhodotorula*, *Wickerhamia*, and *Rhodosporidium* grew in a medium containing 10 mL·L^−1^ of motor oil [9]. Moreover, *Candida tropicalis* and *Rhodosporidium toruloides* assimilated various alkanes (C8–C39) of WMO, with *C. tropicalis* also metabolizing PAH like anthracene, phenanthrene, and benzo(a)pyrene [10].

*Yarrowia lipolytica*, a non-conventional yeast, can be isolated from contaminated environments with crude oil and waste motor oil [11,12], owing to multiple-gene families involved in the metabolic pathways for the efficient degradation of alkanes [13]. In addition, *Y. lipolytica* strains have shown their exceptional lipid accumulation capabilities from a wide range of wastes and by-products, such as waste cooking oils [14], animal fat [15], food-waste-derived volatile fatty acids [16], lignocellulosic biomass hydrolysate [17], and alkanes and alkenes [18,19]. These lipids generally have a fatty acid composition comparable to common vegetable oils, making them a promising raw material for biodiesel production [20]. Furthermore, it was demonstrated that biodiesel produced from *Y. lipolytica* lipids is a high-quality fuel and meets the criteria set by international biodiesel standards [21,22,23].

Biodiesel is a renewable fuel with a biodegradable nature, low sulfur content, favorable carbon footprint, and a ready-to-use mixture in the preexisting engines [24]. The depletion of fossil fuel reserves due to the unprecedented utilization of fossil fuels and economic development led to an extensive search for alternative fuels [25,26]. Biodiesel is commonly obtained from edible vegetable oils (e.g., rapeseed, palm, and soybean oils), competing with the high global demand for these oil feedstocks and arable land for food production. Relying on using vegetable oils for biodiesel manufacturing could lead to a lack of edible vegetable oils, escalating food prices, and creating a conflict between food and fuel priorities [27]. Biodiesel from *Y. lipolytica* lipids is a promising source of bioenergy to reduce the overburden on fossil fuels and does not compete with the arable land for edible oil crops [28]. However, the synthesis of microbial lipids is still associated with high costs of pure substrates, which is considered a major barrier to their commercial-scale production [29].

The potential of WMO as an inexpensive carbon source or co-substrate in *Y. lipolytica* cultures has already been studied [21,30]. However, both studies were evaluated in batch cultures performed at Erlenmeyer flasks, and the yeast growth or lipid yield achieved in those studies is still unsatisfactory. The results described by Miranda et al. [18] showed the ability of *Y. lipolytica* strains CBS 2075 and DSM 8218 to grow and produce lipid-rich biomass and protease from a mixture of alkanes (dodecane and hexadecane) and alkenes (hexadecene), demonstrating the potential of using these two strains to produce lipids and enzymes from hydrocarbon-containing mixtures.

Hence, this work aimed to evaluate the potential of *Yarrowia lipolytica* strains (CBS 2075 and DSM 8218) to grow in WMO and produce valuable compounds, thereby establishing a sustainable approach to managing this hazardous waste. Specifically, the study aimed to assess biomass production, lipid accumulation, and protease production under different WMO concentrations, emphasizing the suitability of *Y. lipolytica* lipids for biodiesel production in alignment with EU standards.

Firstly, the growth of these two *Y. lipolytica* strains in a YNB medium with different WMO concentrations (2 g·L^−1^, 5 g·L^−1^, and 10 g·L^−1^) was evaluated in 96-well microplate experiments. Additionally, the growth of both yeast strains in a WMO-based medium was assessed in bioreactor batch cultures to confirm the results obtained in the microplates. For the first time, a two-stage fed-batch culture approach with the addition of concentrated WMO-medium (1 pulse or 5 pulses) after a first batch phase in a mixture of aliphatic hydrocarbons (hexadecane, dodecane, and hexadecene) was tested for producing high lipid-rich biomass concentration and extracellular protease. The biodiesel properties obtained from *Y. lipolytica* lipids were estimated and compared with international criteria to ensure their suitability as an alternative energy source.

## 2. Materials and Methods

### 2.1. WMO Characterization

WMO was collected from a car repair shop and stored at 5 °C until use. WMO had a blackish-brown to black color, comprised 0.7 g·g^−^^1^ of total lipids, and is composed of the following heavy metals and minerals (mg·kg^−^^1^): Ca—1697 ± 160; P—777 ± 80; Zn—738 ± 81; Mg—244 ± 17; Fe—84 ± 9; Cu—12 ± 2; and Ba—0.3 ± 0.1. The PAH in WMO accounted for 870 mg·kg^−^^1^, and the C10–C40 fraction represented 72.5% of the total petroleum hydrocarbons (TPH) (Table 1).

### 2.2. Yeast Strain Preservation and Inoculum Preparation

Cultures of *Y. lipolytica* CBS 2075 and *Y. lipolytica* DSM 8218, grown overnight in YPD medium (20 g·L^−1^ glucose, 20 g·L^−1^ peptone, and 10 g·L^−1^ yeast extract), were used for the preparation of cryo-stocks as previously described in Miranda et al. [19]. One cryo-stock was used for the pre-inoculum cultures (150 mL of YPD medium), which grew in an orbital incubator at 27 °C and 200 rpm for 16 h.

### 2.3. 96-Wells Microplate Experiments

*Y. lipolytica* CBS 2075 and *Y. lipolytica* DSM 8218 were evaluated for their ability to grow in YNB medium with WMO as a carbon source in microscale experiments performed in 96-well microplates. Each well was filled with 20 µL of the pre-inoculum (to obtain an initial optical density of 0.3), 250 µL of YNB without amino acids and with ammonium sulfate (final concentration of 6.7 g·L^−1^ in the wells), and 20 µL of a WMO-based medium (final concentrations of 2 g·L^−1^, 5 g·L^−1^, or 10 g·L^−1^ in the wells) supplemented with Tween 80 (10% *w*/*w* of WMO). Additional experiments were also carried out: (i) a control experiment with no addition of WMO to the YNB medium in the well; (ii) a control experiment without yeast inoculation to assess the interference of WMO in absorbance due to its oily appearance and dark color (used as the blank for the equivalent inoculated experiment); and (iii) a YNB-medium with 6 g·L^−1^ aliphatic hydrocarbons mixture (2 g·L^−1^ hexadecane, 2 g·L^−1^ hexadecene, and 2 g·L^−1^ dodecane), for comparison purposes since both yeasts were able to assimilate these compounds [18]. The microplates were incubated at 27 °C for 72 h, and the yeast growth was assessed by the optical density (λ = 600 nm) at the beginning (0 h) and the end of the experiments (72 h). The results were expressed as the difference between the absorbance at 72 h and the absorbance at 0 h.

### 2.4. Bioreactor Experiments

All bioreactor experiments were conducted in the DASGIP Parallel Bioreactor System (Eppendorf, Hamburg, Germany) filled with 1.2 L of culture medium. The pH was monitored throughout cultivation time using a pH probe (Mettler Toledo^®^, Columbus, OH, USA) and automatically maintained at 5.5 ± 0.5 by the addition of NaOH 2 M or HCl 2 M. Dissolved oxygen concentration (DOC) was measured by a polarographic oxygen probe (Inpro6820/12/320, Mettler Toledo^®^). Cells of *Y. lipolytica* CBS 2075 and DSM 8218 grew overnight for 16 h in a YPD medium. They were harvested by centrifugation and resuspended in the culture medium at an initial biomass concentration of 0.5 g·L^−1^.

#### 2.4.1. Batch Cultures

The *Y. lipolytica* CBS 2075 and *Y. lipolytica* DSM 8218 growth in different WMO concentrations was evaluated in a culture medium composed of WMO (2 g·L^−1^, 5 g·L^−1^, or 10 g·L^−1^), 3.4 g·L^−1^ corn steep liquor (CSL, C4648-500G, Sigma-Aldrich^®^, St. Louis, MO, USA, mainly composed of (*w*/*w*): total organic carbon—19.5% ± 0.9%; total nitrogen—3.2% ± 0.1%; soluble protein—0.33% ± 0.03%; glucose—2.5% ± 0.1%; fructose—3.22% ± 0.01%; and lactic acid—16.9% ± 0.1%), and 0.5 g·L^−1^ ammonium sulfate. These experiments were conducted at 27 °C for 72 h, using a constant specific airflow rate of 2 vvm and an agitation rate of 700 rpm. Samples were taken to measure the WMO concentration at 0 h and 72 h, and biomass concentration was only quantified at the end of the experiments (72 h).

#### 2.4.2. Two-Stage Pulse Fed-Batch Cultures

Firstly, *Y lipolytica* strains grew in a medium with 6 g·L^−1^ of an aliphatic hydrocarbons’ mixture (2 g·L^−1^ dodecane, 2 g·L^−1^ hexadecane, and 2 g·L^−1^ hexadecene), 0.5 g·L^−1^ ammonium sulfate, and 3.4 g·L^−1^ CSL for 30 h. This batch phase was followed by the addition of (i) one pulse of concentrated medium containing CSL and ammonium sulfate, and WMO in final concentrations of 3.4 g·L^−1^, 0.5 g·L^−1^, and 5 g·L^−1^ in the culture medium, respectively, or (ii) five pulses at 30 h, 54 h, 78 h, 102 h, and 126 h of an equal concentrated medium except for WMO (1 g·L^−1^ each pulse). Two-stage pulse-fed batch cultures were performed at a specific airflow rate of 1 vvm and controlled DOC of 30% by automatic variation of agitation rate between 200 rpm and 500 rpm. At proper intervals, samples were collected to quantify biomass concentration, extracellular enzymes, and microbial lipids. At the end of the experiments, culture samples were also collected to quantify the WMO concentration in the medium.

### 2.5. Analytical Methods

#### 2.5.1. WMO Characterization and Substrate Consumption

The characterization of WMO was carried out by an externally certified laboratory. TPH (C10–C40) were measured using GC with FID detection in agreement with the European standards, ČSN EN ISO 14039 and ČSN EN ISO 16703. PAH was determined by the isotope dilution method using high-resolution gas chromatography coupled with high-resolution mass spectrometry following the United States Environmental Protection Agency Method 429, ISO 11338, and IP 346. The moisture content of WMO was quantified by drying the WMO at 105 °C for 24 h, according to the standard methods from the Association of Official Agricultural Chemists [31]. Heavy metals and minerals were analyzed by the Inductively Coupled Plasma-Optical Emission Spectrometry (Optima 8000, PerkinElmer^®^, Glen Waverley, Australia). The operating conditions were a radio frequency power of 1300 W, argon plasma at 8 L·min^−1^, auxiliary gas at 0.2 L·min^−1^, and nebulizer gas at 0.5 L·min^−1^. The wavelengths (nm) used for each element were: Ba—455.403, Ca—317.933, Cu—327.393, Mg—285.213, Fe—238.204, Zn—213.857, and P—213.617. Total lipids in the WMO were determined gravimetrically, in triplicate, after extraction with chloroform and methanol (adapted from Bligh and Dyer [32]). WMO sample (0.4 g, W1) was mixed with 1.6 mL of distilled water, 4 mL of chloroform, and 2 mL of methanol and vortex-mixed. After filtration, the mixture was transferred to a glass tube previously weighted (W2), and the aqueous phase was collected with a Pasteur glass pipette. The lower phase (organic phase with lipids) was left inside the glass tube in the fume hood until the evaporation of solvents and weighed (W3). The amount of lipids, expressed as the mass of lipids per gram of WMO, was given by the equation: (W3−W2)/W1.

WMO concentration was determined gravimetrically after a liquid-liquid extraction by adding hexane as solvent (2:1, *v*/*v*). The mixture was placed in the orbital incubator at 200 rpm and room temperature for 30 min. After removing the aqueous phase, the organic phase was collected in pre-weight beakers and left in the fume hood until total evaporation of hexane. The combined weight of the beaker and the extracted oil was measured, and the weight of the beaker was subtracted to determine the weight of the oil. All measurements were performed in triplicate.

#### 2.5.2. Yeasts Biomass

Biomass concentration in the 96-well microplate and two-stage pulse-fed batch cultures was determined by measuring the optical density (λ = 600 nm) of the culture samples and converting the absorbance to cell dry weight (g·L^−1^) using a calibration curve for each yeast strain. In batch cultures carried out in the bioreactor, cell dry weight (g·L^−1^) was quantified at the end of the experiments (72 h) after filtration of 5 mL of culture (WMO without cells as blank) through a mixed cellulose esters membrane filter (0.45 µm pore size, Whatman^®^, Maidstone, UK), followed by drying at 60 °C until constant weight.

#### 2.5.3. Extracellular Protease Activity Assay

Extracellular protease activity was quantified in samples supernatant using, respectively, p-nitrophenyl butyrate (1 mM) and 0.5% (*w*/*v*) azocasein as substrates [19].

#### 2.5.4. Microbial Lipids Extraction and Fatty Acid Analysis

Microbial lipid content was quantified in lyophilized cells by the phospho-vanillin colorimetric method after lipid extraction using a chloroform/methanol mixture (1:1) [14]. Before lyophilization, yeast cells were washed with 5 mL of hexane, followed by 5 mL of distilled water to remove potential oil droplets attached to *Y. lipolytica* cells. These results were expressed as lipid content (ratio between lipid concentration and lyophilized biomass concentration) and lipid concentration, calculated by multiplying lipid content by biomass concentration in the cultivation medium.

The fatty acid profiles of microbial lipids were determined by analysis of fatty acid methyl esters using gas chromatography, according to the protocol described by [14]. Hydrocarbons (dodecane, hexadecane, and hexadecene) were extracted through a liquid-liquid extraction, employing hexane as the solvent (1:6, *v*/*v*) ratio and undecane (C11) as the internal standard, as previously described in Miranda et al. [19].

### 2.6. Determination of Biodiesel Properties

Several mathematical equations were applied to estimate the biodiesel properties based on the fatty acid composition of lipids from both *Y. lipolytica* strains in two-stage pulse-fed batch cultures with 5 pulses. Density (ρ), kinematic viscosity (ϑ), saponification value (SV), iodine value (IV), cetane number (CN), higher heating value (HHV), cloud point (CP), oxidative stability (OS), degree of unsaturation (DU), long-chain saturated factor (LCSF), cold filter plugging point (CFPP), and pour point (PP) were calculated using the following equations described in the literature [33,34,35]:

$$\rho \;\left(kg\cdot m^{-3}\right)=\sum N(0.85+\left(\frac{4.9}{MW}\right)+0.0118\times D$$

$$\vartheta \;\left({mm}^{2}\cdot s^{-1}\right)=\sum N\left(-12.5+\left(2.5\times ln\left(MW\right)\right)\right)-0.18\times D$$

$$SV\;\left(mg\cdot g^{-1}\right)=\sum \frac{560\times N}{MW}$$

$$IV\;\left(\frac{mgI_{2}}{100g}\right)=\sum \frac{254\times D\times N}{MW}$$

$$CN=46.3+\frac{5458}{SV}-\frac{0.23}{IV}$$

$$HHV\;\left(MJ\cdot {kg}^{-1}\right)=\sum N\left(46.19-\left(\frac{1794}{MW}\right)-0.21\times D\right)$$

$$CP\;\left({}^{\circ}C\right)=\left(0.526\times C_{16:0}\right)-4.992$$

$$OS\;\left(h\right)=\frac{117.93}{\%C18:2}+2.6$$

$$DU=\sum \left(\%C16:1+\%C17:1+\%C18:1\right)+\left(2\times \%C18:2\right)$$

$$LCSF=\left(0.1\times \%C16:0\right)+\left(0.5\times \%C18:0\right)$$

$$CFPP\;\left({}^{\circ}C\right)=\left(3.14\times LCSF\right)-16.5$$

$$PP\;\left({}^{\circ}C\right)=\left(0.57\times \%C16:0\right)-12.2$$

where N, D, and MW indicate the percentage, number of double bonds, and molecular weight of each fatty acid, respectively.

### 2.7. Statistical Analysis

Statistical analysis was performed with Statgraphics Centurion XVI Version 16.2.04 (StatPoint Technologies Inc., Warrenton, VA, USA), using Tukey’s multi-range test and one-way analysis of variance. A confidence level of 95% was selected for the statistical analysis.

## 3. Results and Discussion

### 3.1. 96-Wells Microplate Experiments

The WMO used in this work is a complex mixture mainly composed of hydrocarbons from C10 to C40 (72.5%) and naphthalene, phenanthrene, and fluorene as the major compounds in the PAH fraction (Table 1).

**Figure 1:**
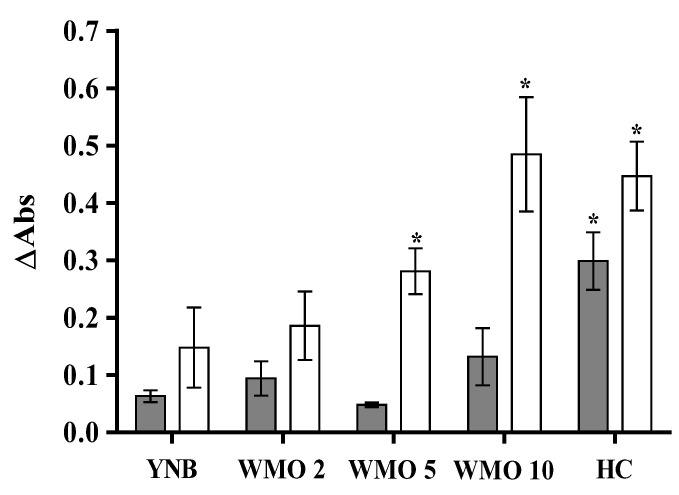
*Yarrowia lipolytica* CBS 2075 (grey bars) and DSM 8218 (white bars) growth in YNB medium (control), YNB medium + WMO (2 g·L^−1^, 5 g·L^−1^, and 10 g·L^−1^), and YNB medium + hydrocarbon mixture 6 g·L^−1^ (HC) evaluated by the difference in the optical density (λ = 600 nm) at 72 h and the beginning of experiments. The error bars represent the standard deviation of six independent replicates. Bars with the asterisk (*) present statistically significant differences from the control (YNB medium) (*p* ˂ 0.05).

Although *Y. lipolytica* CBS 2075 showed limited cell growth in media with WMO, its presence did not inhibit yeast growth, regardless of the concentration. This strain growth in cultures supplemented with WMO was statistically equal to that reached in the YNB medium without WMO (Figure 1). At 10 g·L^−1^ of WMO, the growth of the CBS 2075 strain was slightly higher than that obtained in the YNB medium (control), even though the difference was not statistically significant.

*Y. lipolytica* DSM 8218 was more efficient in WMO assimilation than the other strain. In cultures with 5 g·L^−1^, *Y. lipolytica* DSM 8218 growth was 2-fold and 6-fold higher than that obtained in the YNB medium and with the strain CBS 2075, respectively. Moreover, with 10 g·L^−1^ WMO, growth of DSM 82183 was 3-fold and 4-fold higher than that reached in the YNB medium and CBS 2075 cultures, respectively, demonstrating the high capacity of this strain to assimilate and grow in WMO, including at the highest oil concentrations.

*Y. lipolytica* DSM 8218 was isolated from a diesel storage tank, and it is expected that this yeast strain is adapted to hydrocarbon-rich environments, including high hydrocarbon concentrations, potential microbial inhibitors, and possible environmental fluctuations such as low dissolved oxygen concentrations and pH levels. According to Cerniglia and Crow [36], this *Y. lipolytica* strain was able to metabolize aromatic hydrocarbons such as naphthalene, biphenyl, and benzo(a)pyrene. Other authors reported that *Y. lipolytica* DSM 8218 could metabolize cyclic alkanes resulting from depolymerized polypropylene by pyrolysis [37]. Similarly, *Y. lipolytica* IMUFRJ 50682 was able to degrade naphthalene and phenanthrene from crude oil [38,39]. In contrast, *Y. lipolytica* strains PG-20 and PG-32 did not grow in cultures supplemented with naphthalene, phenanthrene, and pyrene when compared to aliphatic hydrocarbons (C11–C16) [11].

Both *Y. lipolytica* strains were capable of growing in the hydrocarbon mixture (HC), which follows the results of Miranda et al. [18]. Nevertheless, the growth of DSM 8218 in the HC mixture was 1.5-fold higher than that reached with the strain CBS 2075 (Figure 1).

### 3.2. Bioreactor Experiments

#### 3.2.1. Batch Cultures

To validate the ability of the *Y. lipolytica* strains CBS 2075 and DSM 8218 to grow in a medium containing up to 10 g·L^−1^ of WMO, batch cultures were performed at a lab-scale stirred tank bioreactor with controlled pH and aeration rate.

In the bioreactor, it was possible to reproduce the overall results obtained in the microplate well experiments for *Y. lipolytica* CBS 2075. This strain was able to grow and assimilate WMO in cultures with 2 g·L^−1^, achieving a final biomass concentration of 1 g·L^−1^, while 0.65 g·L^−1^ of WMO was assimilated by the yeast (Table 2). Still, the increase of WMO above 2 g·L^−1^ reduced the yeast growth and WCO assimilation without statistically significant differences (*p* > 0.05) between the experiments with 5 g·L^−1^ and 10 g·L^−1^ of WMO. A similar biomass yield was obtained for all WMO concentrations, which were overall higher than those attained with the DSM 8218 strain (Table 2). In CBS 2075 experiments, the final biomass concentrations were similar or even slightly higher than in the microplate, whose final biomass concentration ranged from 0.14 g·L^−1^ and 0.31 g·L^−1^.

Regarding DSM 8218 cultures, the final biomass concentration, the WMO consumption, and biomass yield attained in the bioreactor were statistically equal for all concentrations of WMO (Table 2). Moreover, the final biomass obtained in the bioreactor for all WMO concentrations was higher than that obtained in the microplate experiments, ranging from 0.33 g·L^−1^ and 0.86 g·L^−1^.

In an oil-in-water system, access of the cells to the oily substrate depends on the stability of the emulsion. In bioreactor experiments, better dispersion of the WMO in the culture medium was achieved in the bioreactor due to the high agitation rate employed (up to 700 rpm), which facilitates the contact between the yeast cells and the oil droplets. Taking this into account, it is not surprising the higher growth of both strains in the bioreactor experiments, especially the DSM 8218. Miranda et al. [19] demonstrated that the increase in agitation rate in the bioreactor enhanced the growth of *Y. lipolytica* CBS 2075 in hexadecane-based cultures.

#### 3.2.2. Two-Stage Pulse Fed-Batch Cultures

Since both *Y. lipolytica* strains were not able to assimilate all WMO added to the media, a two-stage pulse-fed batch strategy was tested, aiming to adapt yeast cells to hydrocarbons and initiate the WMO-based bioprocess with a higher biomass concentration than in the batch experiments. It was previously demonstrated that both *Y. lipolytica* CBS 2075 and DSM 8218 strains can effectively assimilate dodecane, hexadecane, and hexadecene with high biomass/substrate yields [18]. This first phase was followed by the addition of 1 pulse or 5 pulses of WMO-concentrated medium at specific time points. The importance of high-density cultures in the assimilation of crude oil by yeasts was reported by Martins et al. [40], who found that a high percentage of alkanes and PAH (>90%) was assimilated by *Y. lipolytica* IMUFRJ 50682 in the culture with the highest initial biomass concentration. Cultures with high initial yeast cell concentration were more efficient in the assimilation of hydrocarbons from the crude oil, including in experiments without nutrient supplementation [38].

**Figure 2:**
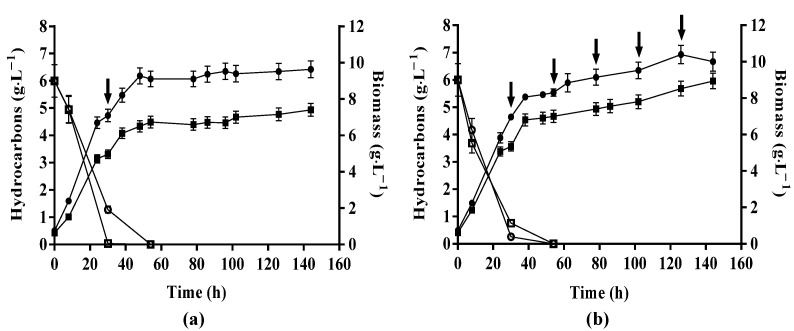
Biomass concentration (closed symbols) and total hydrocarbon consumption (open symbols) obtained in two-stage pulse-fed batch cultures of *Y. lipolytica* CBS 2075 (●, ○) and *Y. lipolytica* DSM 8218 (■, □) carried out with 1 pulse (3.4 g·L^−1^ CSL, 0.5 g·L-1 ammonium sulfate, and 5 g·L^−1^ WMO) (**a**) and 5 pulses (3.4 g·L^−1^ CSL, 0.5 g·L^−1^ ammonium sulfate, and 1 g·L^−1^ WMO) (**b**) of WMO-concentrated medium. The error bars represent the standard deviation of two independent replicates. The arrows indicate the time at which a pulse of WMO-concentrated medium was added to the culture.

In experiments with 1 pulse, the biomass concentration reached in *Y. lipolytica* CBS 2075 and DSM 8218 cultures was approximately 7 g·L^−1^ and 5 g·L^−1^, respectively, after 30 h of growth in the HC mixture. Moreover, all three aliphatic hydrocarbons have already been assimilated by both yeasts at the time of the pulse (30 h) (Figure 2a), which is per the results of Miranda et al. [18]. Adding 1 pulse of 5 g·L^−1^ WMO-concentrated medium did not affect the cells’ viability since both yeast strains continued to grow and entered a new exponential growth phase after the pulse that lasted until 48 h, followed by a stationary stage (Figure 2a). After the pulse addition, biomass produced by CBS 2075 and DSM 8218 from WMO (between 30 h and 144 h of cultivation) was 5-fold and 3.5-fold higher, respectively, than the values obtained in batch cultures (Table 2), demonstrating the importance of yeast cultures with an initial high density to obtain enhanced cell growth and WMO assimilation. Although biomass production rates obtained after the pulse in CBS 2075 (0.12 g·L^−1^·h^−1^ ± 0.01 g·L^−1^·h^−1^) and DSM 8218 (0.084 g·L^−1^·h^−1^ ± 0.004 g·L^−1^·h^−1^) cultures were, approximately, 2-fold lower than those values reached during the batch phase (30 h), an increase of 2-fold in the biomass production rate was attained for CBS 2075 herein compared to batch cultures with 5 g·L^−1^ WMO (Table 2).

Due to the high cell densities at the time of the pulse addition, a higher concentration of WMO was also assimilated by both strains compared to that in the batch cultures (Table 2). A 5-fold and 1.7-fold increase in the concentration of WMO consumed by CBS 2075 and DSM 8218, respectively, was obtained in experiments with a pulse addition (Table 3). Despite that, no significant enhancement in the global biomass yields of CBS 2075 and DSM 8218 was achieved between experiments with 1 pulse (Table 3) and batch cultures (Table 2). In CBS 2075 cultures, biomass production was higher than that attained by the DSM 8218 strain (Figure 2a), but no significant differences were found in the concentration of WMO assimilated and global biomass yields achieved for both strains in experiments with 1 pulse (Table 3).

In the experiments with 5 pulses, regardless of the *Y. lipolytica* strain, the sequential addition of 1 g·L^−1^ WMO-concentrated medium favored continuous cell growth, preventing both strains from reaching a stationary stage (Figure 2b). In CBS 2075 cultures, a 6-fold and 1.2-fold improvement in biomass production from WMO (between 30 h and 144 h of cultivation) was obtained in experiments with 5 pulses relative to biomass produced in batch cultures and experiments with 1 pulse, respectively. With the DSM 8218 strain, biomass produced was, respectively, 5-fold and 1.5-fold higher than those obtained in batch cultures with 5 g·L^−1^ WMO (Table 2) and experiments with 1 pulse (Figure 2a).

Similar concentrations of WMO were consumed by both strains with 5 pulses, which were also statistically equal to those obtained with a single pulse (Table 3). Nevertheless, a 5-fold and 1.8-fold increase in the concentration of WMO consumed by CBS 2075 and DSM 8218 was obtained with 5 pulses, respectively, when compared to batch cultures with 5 g·L-1 WMO (Table 2). Similar global biomass yields were attained in the experiments with 5 pulses for both strains (Table 3), which were also statistically equal to those obtained with 1 pulse. Moreover, no significant differences were found for the global biomass yield obtained in experiments with 5 pulses (Table 3) and batch experiments with 5 g·L^−1^ WMO (Table 2) in CBS 2075 cultures, while the global biomass yield of DSM 8218 in experiments with 5 pulses was 2-fold higher than that obtained in batch cultures (Table 2).

Overall, these results suggest that two-stage pulse-fed batch cultures with either 1 pulse of 5 g·L^−1^ WMO or 5 pulses of 1 g·L^−1^ WMO are advantageous strategies to improve biomass production and WMO assimilation by both strains. Moreover, adding 5 g·L^−1^ WMO in 5 pulses promoted continuous yeast growth, in contrast to what occurs in the experiments with a single pulse. Thus, by adding a lower concentration with each pulse, the inhibitory effects of WMO may be reduced, allowing the yeasts to adapt to the new conditions and continue growing.

From an industrial point of view, proteases are important enzymes since they can break peptide bonds from proteins. These enzymes have several applications in chemical and biochemical processes, mostly used in the detergent, pharmaceutical, food, and wastewater treatment industries [41]. Previous results showed high proteolytic activity in cultures of *Y. lipolytica* CBS 2075 and DSM 8218 growing in hexadecane [19] and an HC mixture [18]. To the best of our knowledge, this is the first work reporting protease production by *Y. lipolytica* strains in a WMO medium.

Both strains produced protease in a medium with HC mixture (first 30 h of cultivation) in all strategies. However, maximum proteolytic activities shown in Table 3 for experiments with 1 pulse and 5 pulses were attained at 48 h and 144 h, respectively, which indicates that both strains continue to produce protease during the pulse-fed batch phase by assimilating WMO since hydrocarbons had already been consumed by yeasts.

Two main profiles of protease production were observed for both yeast strains, depending on pulse strategy: (i) in the experiments with 1 pulse, protease activity reached a maximum after 48 h and 54 h in *Y. lipolytica* CBS 2075 and DSM 8218 cultures, respectively, remaining constant until the end of the cultivation; (ii) with 5 pulses, protease activity progressively increased, attaining a maximum activity at the end of cultivation time (144 h). In the experiments with 1 pulse of 5 g·L^−1^ WMO-concentrated medium, proteolytic activity obtained in *Y. lipolytica* CBS 2075 cultures was 3-fold higher than in DSM 8218 cultures (Table 3). By contrast, in the experiments with 5 pulses of 1 g·L^−1^ WMO-concentrated medium, no significant differences were found for protease produced by both strains. Whereas protease activity obtained in experiments with 1 pulse or 5 pulses was statistically equal for the CBS 2075 strain, protease produced by DSM 8218 in experiments with 5 pulses was 3-fold higher than that obtained in experiments with 1 pulse. Thus, the results suggest that a two-stage pulse-fed batch culture with 5 pulses of 1 g·L*^−^*^1^ WMO-concentrated medium can be an advantageous strategy to improve protease production, particularly in *Y. lipolytica* DSM 8218 cultures. It is worth noticing that the proteolytic activities reached in this study with the CBS 2075 strain and with the DSM 8218 in experiments with 5 pulses were similar or even higher than those achieved in cultures with 5 g·L*^−^*^1^ of hexadecane [19].

The accumulation of intracellular lipids by *Y. lipolytica* from hydrophobic substrates (e.g., fatty acids, triacylglycerols, and alkanes) is a growth-coupled anabolic process known as the *ex novo* pathway, in which the synthesis of lipids occurred simultaneously with the production of lipid-free biomass [42]. Particularly for alkanes, after their uptake by the yeast cells, fatty alcohols are oxidized to fatty acids and then activated to form acyl-CoA. Acyl-CoA undergoes the β-oxidation pathway to produce acetyl-CoA, which can be incorporated in the glyoxylate, Krebs, or methyl-citrate cycles. Alternatively, fatty acids derived from long-chain alkanes (C14–C18) may be stored as triacylglycerols or steryl esters in lipid bodies [13,43].

**Figure 3:**
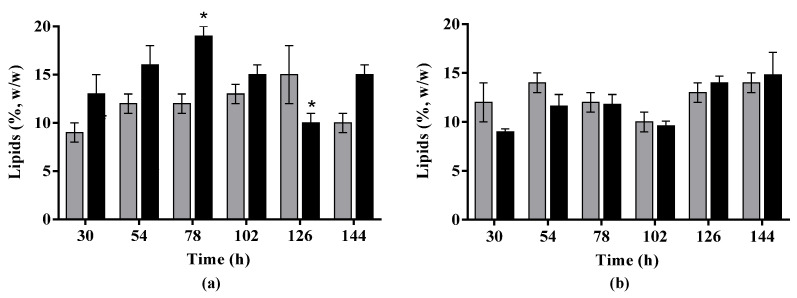
Lipid content of *Y. lipolytica* CBS 2075 (**a**) and *Y. lipolytica* DSM 8218 (**b**) obtained in two-stage pulse-fed batch cultures with 1 pulse (grey black bars) and 5 pulses (black bars) of WMO-concentrated medium. The error bars represent the standard deviation of two independent replicates. Bars with an asterisk (*) for each strategy present statistically significant differences (*p* ˂ 0.05).

Slightly higher lipid contents were reached by adding 5 pulses of 1 g·L^−1^ WMO-concentrated medium instead of a single pulse of 5 g·L^−1^ in CBS 2075 cultures, despite the difference only being statistically significant at 78 h (maximum lipid content of 19%, *w*/*w*) and 126 h (minimum lipid content of 10%, *w*/*w*) (Figure 3a). In cultures of DSM 8218, no significant differences were found in lipid content obtained with 1 or 5 pulses of WMO-concentrated medium (Figure 3b). Moreover, no significant mobilization of lipids was observed in both strategies for both *Y. lipolytica* strains, indicating that two-stage pulse-fed batch cultures with a single pulse of 5 g·L^−1^ WMO or 5 pulses of 1 g·L^−1^ WMO are efficient strategies to improve lipid biosynthesis without significant lipid mobilization during cultivation.

Regardless of the yeast strain, the addition of a WMO-concentrated medium (1 pulse of 5 g·L^−1^ or 5 pulses of 1 g·L^−1^ WMO-concentrated medium) led to a significant improvement in the lipid content compared to experiments without WMO, particularly after 30 h (Figure 3a,b), suggesting that the addition of WMO using pulses favored lipid accumulation by *Y. lipolytica* strains. In contrast, Magdouli et al. [30] reported that the presence of some additives in WMO, such as synthetic antioxidants and corrosion inhibitors, negatively affected cell growth and the synthesis of lipids by *Y. lipolytica* strains in a crude glycerol-based medium. Indeed, several additives may be present in WMO, such as amide and amine derivatives, methyl esters, polymethacrylate, oxygen- and metal-containing compounds, and phenols. These compounds are incorporated into new motor oils to function as, for example, antioxidants, foam inhibitors, viscosity and friction modifiers, detergents, dispersants, pour-point depressants, and thermal property enhancers [44].

Regardless of the yeast strain, the concentrations of lipids achieved in experiments with 1 pulse or 5 pulses were statistically equal (Table 3). Despite the difference being not statistically significant, the addition of a single pulse of 5 g·L^−1^ or 5 pulses of 1 g·L^−1^ WMO-concentrated medium led to, respectively, a 2-fold and 2.5-fold increase in lipid concentration obtained by both strains compared to experiments without WMO. From an operational perspective, a single addition would be more advantageous for lipid production in an industrial context. Herein, both strategies, 1 pulse or 5 pulses, proved to be promising approaches for lipid production by *Y. lipolytica* strains in cultures containing WMO.

It is important to highlight that the lipids produced by CBS 2075 and DSM 8218 strains in 5 pulses of WMO-concentrated medium were, respectively, 3.5-fold and 2.7-fold higher than the values obtained in cultures of *Y. lipolytica* NCIM 3589 growing in WMO as reported by Katre et al. [21]. Although *Y. lipolytica* CBS 2075 and DSM 8218 strains accumulated lower quantities of lipids when compared to those reported in cultures with oil-rich wastes such as pork lard [15], waste cooking oils [14,45], chicken tallow [46], and fish waste oil [47], this study is one of the first works demonstrating the potential of using WMO for the accumulation of lipids by *Y. lipolytica* strains.

In general, lipids synthesized by *Y. lipolytica* CBS 2075 and DSM 8218 in two-stage fed-batch cultures were mainly composed of unsaturated fatty acids (59–68%), regardless of the pulses’ strategy. By contrast, strains of *Y. lipolytica* (NCIM 3529, NCIM 3450, NCIM 3472, and NCIM 3590) accumulated highly saturated lipids (70–90%) from WMO [21], suggesting that lipid accumulation in *Y. lipolytica* is a strain-related mechanism. Lipids of Y*. lipolytica* CBS 2075 and DSM 8218 were mainly composed of stearic (C18:0), oleic (C18:1), and linoleic (C18:2) acids in similar percentages, followed by palmitic (C16:0) and palmitoleic (C16:1) acids (Table 4). It is worth noticing that a high content of stearic acid (C18:0) was reached, approximately 20%, in contrast to lipids accumulated by *Y. lipolytica* strains CBS 2075 and DSM 8218 from hexadecane and an HC mixture (hexadecane, hexadecene, and dodecane), in which no stearic acid [19] or significant lower quantities (3–5%) [18] were synthesized. Lipids of *Y. lipolytica* strains from other carbon sources, such as pork fat [15], waste cooking oils [14,21], and volatile fatty acids [48,49,50], were richer in oleic and linoleic acids.

Regarding lipids accumulated by *Y. lipolytica* CBS 2075, no significant differences were found between the fatty acids’ compositions for both pulse strategies. In cultures with 1 pulse, the CBS 2075 strain accumulated heptadecenoic acid (C17:1), though in small amounts (Table 4). Heptadecenoic acid is an odd-chain fatty acid known to benefit the quality of the biodiesel obtained from *Y. lipolytica* lipids, improving transesterification reactions or storage conditions [51]. In general, lipids accumulated by *Y. lipolytica* DSM 8218 showed a similar fatty acid composition for both pulse strategies, except for palmitic acid (C16:0), whose content was 1.5-fold higher in experiments with 5 pulses than in experiments with a single pulse (Table 4).

The fatty acid profile is one of the major parameters affecting biodiesel quality and its properties, and to obtain high-quality biodiesel, saturated and unsaturated fractions must have an optimal balance [33,52]. Lipids accumulated by *Y. lipolytica* CBS 2075 and DSM 8218 closely resemble vegetable oils, the common raw material used in the biodiesel industry [20]. The quality of biodiesel is influenced by multiple parameters, including density, kinematic viscosity, heating value, cetane number, oxidation stability, iodine value, and cold flow properties. These characteristics are essential for evaluating biodiesel’s viability as a substitute for petrodiesel [53]. The fatty acid profile of the lipids used, specifically the degree of unsaturation, the number and position of double bonds, and the fatty acid chain length, significantly affect biodiesel properties [53,54].

In this study, a variety of biodiesel parameters were calculated based on the fatty acid profiles of lipids accumulated by both *Y. lipolytica* strains in two-stage fed-batch cultures with the addition of 5 pulses of WMO-concentrated medium (Table 5). The key parameters density, kinematic viscosity, cetane number, oxidation stability, and iodine value agreed with the criteria defined by the EU biodiesel standard EN 14214 [55], making the synthesized lipids a potential feedstock for biodiesel production.

In the literature, three fundamental parameters define the cold flow properties of fuels: cloud point, pour point, and cold filter plugging point (CFPP). Whereas the cloud point indicates the lowest temperature before the least soluble components of fuel start crystallizing, the pour point is the lowest temperature at which the fuel remains fluid [56]. Among them, CFPP is the most useful and commonly evaluated. This parameter indicates the lowest temperature (°C) at which biodiesel can pass through a standardized filter within a certain time without causing plugging problems [53]. A lower CFPP is linked to better cold flow properties, which are crucial in colder climates to prevent clogged fuel lines, filters, and injectors [56]. Since CFPP is highly dependent on season and temperature, no limits have been specified in the EU biodiesel standard EN 14214 for this parameter. Still, in this work, CFPP values ranged between 20 °C and 22 °C, agreeing with the broad range of values reported for biodiesel obtained from other *Y. lipolytica* strains (Table 5).

Kinematic viscosity and the cetane number of the fuel are the other two important parameters to take into consideration. Kinematic viscosity refers to biodiesel’s flow characteristics and will influence ignition and fuel injection quality and quantity. Higher viscosity can lead to performance issues, particularly at low temperatures, whereas lower viscosity results in thin fuel particles with high speed and low mass [53]. On the other hand, the cetane number indicates the self-ignite quality of the biodiesel and is strongly related to ignition delay time. It affects the overall engine performance, including stability, noise levels, and carbon monoxide emissions [55]. High levels of saturated fatty acids lead to a higher cetane number, enhancing ignition quality and oxidative stability but poor cold flow properties. By contrast, unsaturated fatty acids, particularly polyunsaturated, improve overall biodiesel’s performance at low temperatures but result in a lower cetane number and decreased oxidative stability, making them less favorable for biodiesel manufacturing [33,56]. Herein, both the kinematic viscosity and cetane number of biodiesel obtained from lipids of *Y. lipolytica* CBS 2075 and DSM 8281 are consistent with the standards established by the EU and with other works reported in the literature for biodiesel from *Y. lipolytica* lipids (Table 5) and biodiesel produced from common vegetable oils [56].

The high heating value represents the energy released during combustion, directly relating to the biofuel’s efficiency. Though there are no specific recommendations for high heating value, the HHV determinate herein, approximately 40 MJ·kg^−1^ (Table 5), agreed with the range of values reported in the literature for biodiesel obtained from lipids of *Y. lipolytica* strains cultivated on waste fats [23,46] and waste cooking oil [21].

The oxidative stability of a fuel is also a relevant criterion for the determination of biodiesel quality. In this work, oxidative stability meets the limits established by EU biodiesel standard EN 14214 (Table 5). The degree of unsaturation, as well as the number and location of double bonds in fatty acid chains, will affect negatively biodiesel oxidative stability and its long-term storage. As biodiesel is oxidized, viscosity and cetane number increase due to gum formation and shorter ignition delay, while iodine value tends to decrease [56]. Other major parameters, density, and iodine value agreed with the criteria by EU biodiesel standard EN 14214 [55]. In summary, the estimated biodiesel properties obtained herein demonstrated that biodiesel obtained from lipids of *Y. lipolytica* CBS 2075 and DSM 8218 is highly suitable for producing a high-quality biofuel.

## 4. Conclusions

This study is the first work reporting the protease and intracellular lipid production by *Y. lipolytica* in waste motor oil (WMO)-based medium, highlighting the potential of *Y. lipolytica* CBS 2075 and DSM 8218 strains to valorize WMO. Batch experiments in a microplate and stirred-tank bioreactor demonstrated the ability of both strains to grow in different WMO concentrations. A two-stage fed-batch culture involving an initial growth phase on aliphatic hydrocarbons followed by WMO-concentrated medium addition in pulses was an effective strategy to improve WMO assimilation and biomass production. Both strains produced protease during the pulse-fed batch phase from WMO, which is a new finding. Intracellular lipids produced were rich in unsaturated fatty acids, making these lipids a plausible feedstock for high-quality biodiesel production. These findings open new perspectives on developing a greener and environmentally friendly bioprocess, focusing on the management of WMO and its bioconversion into valuable compounds by *Y. lipolytica*, in agreement with the bioeconomy and biorefinery assumptions.

## Figures and Tables

**Table 1:** Total petroleum hydrocarbons (TPH) and main polycyclic aromatic hydrocarbons (PAH) of WMO. All values are presented with a measurement uncertainty of 30% (95% confidence level).

Total petroleum hydrocarbons (TPH) (C10–C40 fraction) (%, *w*/*w*)	72.5
Polycyclic aromatics hydrocarbons (PAH) fraction (mg·kg^−1^)	870
Naphthalene	190
Phenanthrene	170
Fluorene	130
Pyrene	74
Anthracene	71
Fluoranthene	41

**Table 2:** Final biomass, WMO consumed, and biomass yield (*Y*_x/s_) obtained at 72 h in *Y. lipolytica* CBS 2075 and DSM 8218 batch cultures in a bioreactor with 2 g·L^−1^, 5 g·L^−1^, and 10 g·L^−1^ of WMO. Data are the average ± standard deviation of two independent replicates. Values followed by the same letter in each column for each strain do not present statistically significant differences (*p* ≥ 0.05).

*Y. lipolytica* Strain	WMO (g·L^−1^)	Biomass (g·L^−1^)	Total WMO Consumed (g·L^−1^)	*Y*_x/s_ (g·g^−1^)
	2	1.0 ± 0.1 ^a^	0.65 ± 0.05 ^a^	1.5 ± 0.3 ^a^
CBS 2075	5	0.5 ± 0.1 ^a^	0.5 ± 0.2 ^a^	0.99 ± 0.05 ^a^
	10	0.50 ± 0.04 ^a^	0.5 ± 0.1 ^a^	1.0 ± 0.1 ^a^
	2	0.7 ± 0.1 ^a^	0.9 ± 0.1 ^a^	0.7 ± 0.1 ^a^
DSM 8218	5	0.7 ± 0.1 ^a^	1.3 ± 0.1 ^a^	0.58 ± 0.03 ^a^
	10	1.4 ± 0.3 ^a^	2.3 ± 0.5 ^a^	0.6 ± 0.2 ^a^

**Table 3:** Total WMO consumed, biomass yield (*Y_X/S_*), protease activity (Prot_max_), and lipid concentration (Lipids_max_) obtained in two-stage pulse-fed batch cultures of *Y. lipolytica* CBS 2075 and *Y. lipolytica* DSM 8218 carried out with 1 pulse with 5 g·L^−1^ of WMO-concentrated medium or 5 pulses with 1 g·L^−1^ of WMO-concentrated medium. Data are the average ± standard deviation of two independent replicates. The value in column “Prot_max_” for the DSM 8218 strain, followed by the asterisk (*), presents statistically significant differences (*p* ≥ 0.05).

*Y. lipolytica* Strain	Strategy	Total WMO Consumed (g·L^−1^)	Y_x/s_ (g·g^−1^)	Prot_max_ (U·L^−1^)	Lipids_max_ (g·L^−1^)
CBS 2075	1 pulse	2.5 ± 0.2	1.1 ± 0.3	581 ± 29	1.4 ± 0.2
	5 pulses	2.4 ± 0.4	1.1 ± 0.4	628 ± 37	1.7 ± 0.3
DSM 8218	1 pulse	2.2 ± 0.1	0.8 ± 0.3	204 ± 10	1.05 ± 0.01
	5 pulses	2.3 ± 0.2	1.1 ± 0.4	593 ± 30 *	1.3 ± 0.3

*Y*_x/s_ was expressed as the mass of cells per mass of hydrocarbons and WMO consumed in total.

**Table 4:** Fatty acid composition of microbial lipids produced by *Y. lipolytica* CBS 2075 and Y*. lipolytica* DSM 8218 in two-stage fed-batch cultures with pulses of concentrated WMO. Data are the average ± standard deviation of two independent replicates. Values followed by the same letter in each column for each strain do not present statistically significant differences (*p* ≥ 0.05).

*Y. lipolytica* Strain	Strategy	Relative Fatty Acid Content (%)							
		C16:0	C16:1	C17:1	C18:0	C18:1	C18:2	UFA (%)	SFA (%)
CBS 2075	1 pulse	16 ± 2 ^a^	9.2 ± 0.2 ^a^	5.4 ± 0.2 ^a^	19 ± 1 ^a^	25 ± 1 ^a^	25 ± 1 ^a^	64 ± 3 ^a^	36 ± 2 ^a^
	5 pulses	21 ± 4 ^a^	8 ± 2 ^a^	n.d	20 ± 1 ^a^	25 ± 2 ^a^	26 ± 2 ^a^	59 ± 1 ^a^	41 ± 3 ^a^
DSM 8218	1 pulse	11 ± 2 ^a^	12 ± 1 ^a^	n.d	21 ± 1 ^a^	27 ± 1 ^a^	28 ± 1 ^a^	68 ± 1 ^a^	32 ± 1 ^a^
	5 pulses	17 ± 3 ^b^	11.9 ± 0.3 ^a^	n.d	20 ± 1 ^a^	25 ± 1 ^a^	27 ± 1 ^a^	64 ± 3 ^a^	36 ± 1 ^a^

n.d—not detectable; UFA—total of unsaturated fatty acids; SFA—total of saturated fatty acids.

**Table 5:** Estimated properties of biodiesel obtained from lipids of *Y. lipolytica* CBS and *Y. lipolytica* DSM 8218 and comparison with biodiesel properties obtained from lipids produced by other *Y. lipolytica* strains and the EU biodiesel standard EN 14214.

Properties	*Y. lipolytica* CBS 2075	*Y. lipolytica* DSM 8218	Biodiesel from *Y. lipolytica* Strains [1,2,3,4]	EU Biodiesel Standard EN 14214 [5]
Density (kg·m^−3^)	874	883	856–1019	860–900
Kinematic viscosity (mm^2^·s^−1^)	4	4	3.6–6.4	3.5–5.0
Cetane number	56	54	53–64	51 min
Higher heating value (MJ·kg^−1^)	39	40	37–41	NS
Cloud point (°C)	6	4	11–17	NS
Oxidative stability (h)	7	7	7–33	6 min
Iodine value (mg I_2_/100 g)	78	83	30–66	120 max
Degree of unsaturation	85	91	7.4	NS
Cold filter plugging point (°C)	22	20	−9.3–31	NS *
Pour point (°C)	−0.2	−3	5–12	NS
Saponification value (mg·g^−1^)	204	206	167–256	NS

NS—not specified; NS * CFPP limits depend on geography and time of the year; min—minimum; max—maximum.

## Data Availability

Data are contained within the article.
